# Supplementary material for: Vocal correlates of individual sooty mangabey travel speed and direction
Source: PeerJ. 2016 Jul 28;4:e2298. doi: 10.7717/peerj.2298 (PMC4987050; doi:10.7717/peerj.2298)
Supplement: Supplemental Information 1 [file peerj-04-2298-s001.pdf]

Supplementary material for 'Vocal regulation of individual sooty mangabey travel speed and direction'

Christof Neumann and Klaus Zuberbühler

April 7, 2016

Methods and results

Study species and site

Between April and July 2014, we followed adult and adolescent individuals of a group of sooty mangabeys (*Cercocebus atys*) in Taï National Park, Côte d'Ivoire. All individuals are habituated to close proximity to human observers. In addition to sooty mangabeys, Tai forest is home to six diurnal monkey species (McGraw, Zuberbühler, and Noë, 2007). Sooty mangabeys form poly-specific associations with all of these species regularly. The species is particularly well suited for our study because individuals are often widely spread while visibility on the forest floor is often less than 15 meters (C. Boesch and Boesch, 1984; Janmaat, 2006; Janmaat, Byrne, and Zuberbühler, 2006; Rutte, 1998).

Data collection

We used focal animal sampling (Altmann, 1974) to collect data on randomly selected individuals. The observer followed the individual at close distance, aiming at keeping the distance between observer and animal at around three meters. Audio was recorded continuously, resulting in a single audio file that was analysed later on (see below). During focal follows, the observer spoke the focal individual's behaviour and subgroup size onto the audio recorder. Activity was recorded every minute, subgroup size every two minutes and focal vocalization continuously. We matched time between audio recordings and GPS data using the GPS unit's time as reference setting. Based on the GPS unit's time we set a timer on a wristwatch to signal every 60 seconds with a beep signal. This timer signal was audible on the audio recordings and served as reference point for matching activity, focal behaviour and soundscape. Data collection was stopped if the focal individual was out of sight for more than 60 seconds.

We distinguished three activity types. Feeding/foraging consisted of either ingesting food items, or searching for food. The latter predominantly occurred in the form of an individual standing or sitting on the forest floor using one hand to sift through leaf litter. Travel was defined as continued locomotion over a distance of at least 10 meters, without any active sign of looking for food. Finally, social/resting activity was scored if the individual was engaged in socio-positive behaviour (e.g. grooming), aggression, mating or did not engage in any other behaviour.

Spatial analysis

We recorded GPS points every 30 seconds. Tracks were imported into BaseCamp software (v. 4.4, Garmin Ltd.) and exported as .gpx files. All further processing and calculations were done in R (v.

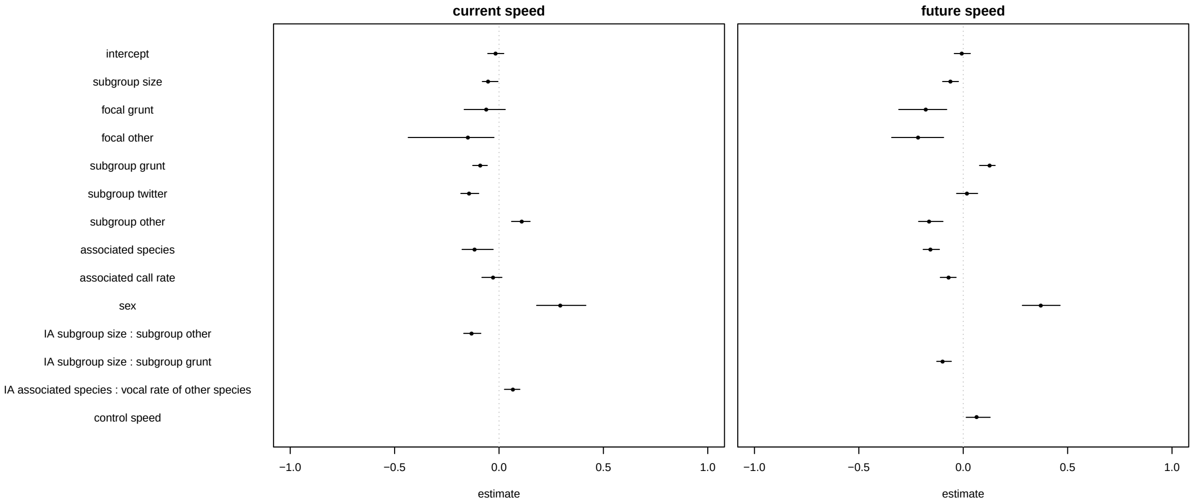

Figure S1: Model estimates (circles) and range of parameter estimates from models for current (left panel) and future (right panel) speed, for which we recalculated our speed variable with added errors as assessed from GPS steps while the GPS unit was stationary. Models were run 100 times.

3.1.1, R Core Team, 2014), using the sp and rgdal packages (Bivand, Keitt, and Rowlingson, 2014; Bivand, Pebesma, and Gomez-Rubio, 2013). We calculated the distance covered in each 5-minute time block as the sum of the distances of the ten steps in each time block (the last step being the step towards the first GPS point of the subsequent time block). We assessed GPS unit precision similarly to Asensio, Brockelman, Malaivijitnond, and Reichard (2011) by letting the units record GPS points at 17 fixed locations in the forest in 30s-intervals and then calculating step lengths and bearing between two consecutive points. This yielded a total of 4,143 distances for each of the two units we used during the study. Mean distance between two points was 0.03m for both units (range: 0.00 – 16.07m). To assess the impact of GPS precision on our results, we refitted our two speed models after modifying the speed (distance over 5-minute time blocks) variables in the following way. Before calculating the total distance covered in a given time block we added to each of the ten GPS fixes per time block a distance and bearing randomly selected from the 4,143 data points obtained from stationary locations. We then recalculated the total distance covered, and thus speed, in each time block as described above. We repeated these steps 100 times for both speed models we fitted and compared fixed effect estimates of the models with randomly added GPS error to our original models. Note that we omitted the auto-correlation term from these models because its calculation requires manual/visual checks of intermediate results (see below). Overall, model results were stable with regards to parameter estimates of interest and we thus concluded that GPS error did not affect our conclusions (Figure S1).

Acoustic analysis

We used ELAN (v. 4.7.3, Brugman and Russel, 2004, Max Planck Institute for Psycholinguistics, The Language Archive, Nijmegen, The Netherlands, http://tla.mpi.nl/tools/tla-tools/elan/) to transcribe the vocal behaviour of the focal individuals and to obtain calling rates from our recordings. If any call overlapped between two subsequent minutes, we assigned the call to the minute in which the larger proportion of the call occurred. Instances when background noise made it impossible to identify calling behaviour (e.g. branch/tree falls, observer walking noisily) were marked as ‘noise’ in ELAN and subsequently removed from calculating calling rates.

Statistical Analysis

Because twitters were never produced by focal individuals during our study, we excluded the variable 'focal individual produced twitter' from our analysis. In all models, we fitted observed individual and observation date as random effects.

We transformed data if necessary to achieve symmetric distributions (log, square root, fourth root) and then standardized variables to mean=0 and standard deviation=1, facilitating interpretation of regression estimates (Schielzeth, 2010). We fitted all models using maximum likelihood rather than restricted maximum likelihood (Bolker et al., 2009).

Model assumptions

We visually inspected histograms of residuals and plotted fitted values against residuals to test assumptions of linear regression. No obvious violations of the assumption of normally distributed and homogeneous residuals were detected. We checked for collinearity by calculating variance inflation factors with the vif function of the car package (Fox and Weisberg, 2011), using linear models without random effects. All variance inflation factors were below 1.63, indicating collinarity not to be an issue (A. Field, Miles, and Field, 2012).

Temporal auto-correlation

Because we used repeated data points per individual that were collected on different dates, we likely violated the assumption for our models of independent residuals (i.e. data points closer in time to each other are more likely to be similar to each other than data points further apart in time). To deal with this potential problem, we calculated an auto-correlation term following procedures developed by Roger Mundry (e.g. Fürtbauer, Mundry, Heistermann, Schülke, and Ostner, 2011). Starting with the residuals from the full model, for each data point we calculated the average of the residuals of all other data points of the same individual. These residuals were weighted by their time lag with respect to the original data point. Following Fürtbauer et al. (2011), the weight was normally distributed with a standard deviation determined by minimizing Akaike's information criterion of the full model that included the term as additional fixed predictor variable.

Effects of behaviour

To test the effects of behaviour on speed, we classified each time block for each individual in the following way. A time block was defined as feeding/foraging if at least four out of the five point samples were 'feeding/foraging' we coded such a time block as 'yes' and as 'no' if this criterion was not met. Likewise, if a time block contained at least one point sample of 'travelling' we coded this as 'yes' and otherwise as 'no'. Similarly, if at least on point sample was either 'resting', 'involved in social activity (grooming/aggression/mating)', this was coded as 'yes' and otherwise as 'no'. We then used univariate Mann Whitney U tests implemented in the exactRankTests package (Hothorn and Hornik, 2013), for which we randomly selected one data point per individual from the data set to circumvent pseudo-replication. We repeated this procedure 1,000 times for each of the three behaviour categories, using current and future speed as response variables. We calculated exact p-values (Mundry and Fischer, 1998) and present averaged results (Table S1).

The results in Table S1 indicate that current speed was only affected behaviourally by whether or not an individual was travelling, which we should have expected. On the other hand, neither feeding/foraging nor social activities were themselves explaining variation in speed. None of the tested behaviours explained variation in future speed or changes in direction. Taken together, this leads us

Table S1: Differences in speed and direction changes depending on current predominant activity. A positive difference indicates that the values were larger in the 'yes' category. For example, current speed tended to be higher during travel time blocks than during non-travel time blocks. Calculations were based on 1000 randomly sampled subsets of data (see text for details).

	n_{no}	n_{yes}	U	difference	p
**current speed**					
feed/forage	3.5	12.5	14.7	−0.32	0.4971
social/rest	11.2	4.8	18.8	0.31	0.5197
travel	12.8	3.2	4.4	1.31	0.0736
**future speed**					
feed/forage	3.5	12.5	16.9	−0.16	0.6200
social/rest	10.6	5.4	18.5	0.48	0.4056
travel	12.8	3.2	15.6	0.25	0.6175
**current direction change**					
feed/forage	3.5	12.5	17.7	−0.02	0.7765
social/rest	11.2	4.8	22.1	−0.02	0.7983
travel	12.8	3.2	16.1	0.09	0.7229
**future direction change**					
feed/forage	3.5	12.5	17.9	0.07	0.7915
social/rest	10.6	5.4	23.7	−0.02	0.7784
travel	12.8	3.2	16.5	0.06	0.7549

to assume that behaviour in itself had no pronounced effect on movement speed or direction changes other than the obvious relationship between travelling and current speed. As feeding/foraging was by far the most common behaviour observed (accounting for 87.9% of point samples) and travelling was rare (2.4% of point samples), we concluded that any effects of travelling on speed could be discounted as large confounders of our models.

Modelling without data points for travelling

To further assess possible confounding effects of "true" travel behaviour, we reran the primary models excluding data points that included at least one point sample for which activity was scored as "travelling" (Table S2). For modelling future speed we additionally excluded data points for which "travelling" occurred in the subsequent 5-minute block (Table S3). In this way, we excluded any confounding covariation between speed and travelling behaviour, which appears to be trivial (during travel blocks speed was higher, see above). In other words, here we only used data that occurred outside the travelling context for which we have no a priori reason to assume a relationship between speed and behaviour.

Overall, we find some consistency as well as some discrepancies between the original models and the ones from which travel was excluded. Our major conclusion that movement coordination is governed by complex interactions between social setting and vocal signals is still supported, as well the notion that vocal rates can accelerate and slow down movements.

However, the effects of associated species' presence and vocalizations on current speed disappeared, though notably the effect was only marginally significant in the original models. Further, in contrast to the original models, there appeared to be effects of the vocalizations of focal animals, i.e. current

speed co-varied with the interactions of subgroup size and focal other vocalizations as well as the interaction between subgroup size and the subgroup's other vocalizations (the latter resembles our findings in the original model).

With regards to future speed, we now see a negative effect of the focal individuals' other vocalizations, while the effect of the subgroup's other vocalizations is still negative. However, as in our original models, the interaction between subgroup size and subgroup grunts still explained variation in future speed.

Table S2: Predictors of current speed, excluding travel-data points.

	$\beta \pm$ se	t	χ^2_1	p
intercept	-0.16 ± 0.90	-0.17		
subgroup size	-0.02 ± 0.05	-0.37		
focal grunt (yes)	-0.18 ± 0.12	-1.55	2.36	0.1243
focal other vocalization (yes)	-0.16 ± 0.14	-1.17		
subgroup grunt	-0.09 ± 0.05	-1.80	3.19	0.0739
subgroup other	0.11 ± 0.05	2.39		
subgroup twitter	-0.19 ± 0.05	-3.58	12.29	0.0005
number of associated species	-0.04 ± 0.08	-0.46	0.20	0.6518
vocal rate of other species	-0.07 ± 0.05	-1.35	1.80	0.1792
sex (male)	2.14 ± 1.71	1.26	1.52	0.2183
auto-correlation	-1.88 ± 0.11	-17.27	109.31	0.0000
IA subgroup size : focal other	-0.53 ± 0.19	-2.84	7.82	0.0052
IA subgroup size : subgroup other	-0.19 ± 0.04	-4.57	19.40	0.0000

Table S3: Predictors of future speed, excluding travel-data points.

	$\beta \pm$ se	t	χ^2_1	p
intercept	0.21 ± 0.67	0.32		
subgroup size	-0.06 ± 0.06	-0.92		
focal grunt (yes)	-0.15 ± 0.15	-1.00	1.00	0.3168
focal other vocalization (yes)	-0.32 ± 0.17	-1.92	3.62	0.0570
subgroup grunt	0.16 ± 0.06	2.60		
subgroup other	-0.13 ± 0.06	-2.24	4.88	0.0272
subgroup twitter	-0.02 ± 0.06	-0.31	0.09	0.7614
number of associated species	-0.03 ± 0.11	-0.31	0.09	0.7679
vocal rate of other species	-0.04 ± 0.07	-0.67	0.43	0.5120
sex (male)	0.43 ± 1.26	0.34	0.11	0.7361
control speed	0.07 ± 0.06	1.26	1.57	0.2095
auto-correlation	-1.59 ± 0.12	-13.66	68.69	0.0000
IA subgroup size : subgroup grunt	-0.12 ± 0.05	-2.21	4.76	0.0291

References

Altmann, J. (1974). Observational study of behavior: sampling methods. *Behaviour*, *49*(3), 227–266. doi:10.1163/156853974X00534

Asensio, N., Brockelman, W. Y., Malaivijitnond, S., & Reichard, U. H. (2011). Gibbon travel paths are goal oriented. *Animal Cognition*, *14*(3), 395–405. doi:10.1007/s10071-010-0374-1

Bivand, R. S., Keitt, T., & Rowlingson, B. (2014). Rgdal: Bindings for the Geospatial Data Abstraction Library (Version 0.9-1). Retrieved from http://CRAN.R-project.org/package=rgdal

Bivand, R. S., Pebesma, E., & Gomez-Rubio, V. (2013). *Applied spatial data analysis with R* (2nd). New York: Springer.

Boesch, C. & Boesch, H. (1984). Mental map in wild chimpanzees: an analysis of hammer transports for nut cracking. *Primates*, *25*(2), 160–170. doi:10.1007/BF02382388

Bolker, B. M., Brooks, M. E., Clark, C. J., Geange, S. W., Poulsen, J. R., Stevens, M. H. H., & White, J.-S. S. (2009). Generalized linear mixed models: a practical guide for ecology and evolution. *Trends in Ecology & Evolution*, *24*(3), 127–135. doi:10.1016/j.tree.2008.10.008

Brugman, H. & Russel, A. (2004). Annotating Multimedia/ Multi-modal resources with ELAN. In *Proceedings of LREC 2004, Fourth International Conference on Language Resources and Evaluation.*

Field, A., Miles, J., & Field, Z. (2012). *Discovering statistics using R.* London: Sage Publications.

Fox, J. & Weisberg, S. (2011). *An R Companion to Applied Regression* (2nd). Thousand Oaks: Sage.

Fürtbauer, I., Mundry, R., Heistermann, M., Schülke, O., & Ostner, J. (2011). You mate, I mate: macaque females synchronize sex not cycles. *PLoS ONE*, *6*(10), e26144. doi:10.1371/journal.pone.0026144

Hothorn, T. & Hornik, K. (2013). exactRankTests: exact distributions for rank and permutation tests, version 0.8-27 (Version 0.8-27). Retrieved March 7, 2015, from http://CRAN.R-project.org/package=exactRankTests

Janmaat, K. R. L. (2006). *Fruits of Enlightenment: Fruit Localization Strategies in Wild Mangabey Monkeys* (PhD thesis, University of St. Andrews, St. Andrews).

Janmaat, K. R. L., Byrne, R. W., & Zuberbühler, K. (2006). Evidence for a spatial memory of fruiting states of rainforest trees in wild mangabeys. *Animal Behaviour*, *72*(4), 797–807. doi:10.1016/j.anbehav.2005.12.009

McGraw, W. S., Zuberbühler, K., & Noë, R. (Eds.). (2007). *Monkeys of the Taï Forest: an African primate community.* Cambridge: Cambridge University Press.

Mundry, R. & Fischer, J. (1998). Use of statistical programs for nonparametric tests of small samples often leads to incorrect *P* values: examples from Animal Behaviour. *Animal Behaviour*, *56*(1), 256–259. doi:10.1006/anbe.1998.0756

R Core Team. (2014). R: A Language and Environment for Statistical Computing. Vienna, Austria.

Rutte, C. (1998). *Strategien der Nahrungssuche bei der Rauchgrauen Mangabe (*Cercocebus torquatus atys*) [Foraging strategies of sooty mangabeys]* (Diplomarbeit, Friedrich-Alexander-Universität, Erlangen/Nürnberg).

Schielzeth, H. (2010). Simple means to improve the interpretability of regression coefficients. *Methods in Ecology and Evolution*, *1*(2), 103–113. doi:10.1111/j.2041-210X.2010.00012.x
