# Supplementary material for: Vocal correlates of individual sooty mangabey travel speed and direction
Source: PeerJ. 2016 Jul 28;4:e2298. doi: 10.7717/peerj.2298 (PMC4987050; doi:10.7717/peerj.2298)
Supplement: Supplemental Information 2 [file peerj-04-2298-s002.pdf]

Data sets and replication code for 'Vocal regulation of individual sooty mangabey travel speed and direction'

Christof Neumann & Klaus Zuberbühler

Data sets

The data for the four models tested in the manuscript are in four separate .csv files. The models are described below in separate sections and refer to:

(1) current speed (dataset1.csv)

(2) future speed (dataset2.csv)

(3) current direction changes (dataset3.csv)

(4) future direction changes (dataset4.csv)

The following variables appear in the data sets:

- `speed` and `controlspeed`: speed of focal individual in a given time block, `controlspeed` is the control variable for the future speed model
- `cp` and `controlcp`: whether or not focal individual's track contained a change point in a given time block, `controlcp` is the control variable for the future speed model
- `subgr.grunt`, `subgr.twitter`, `subgr.other`: vocal rates of mangabeys in the soundscape, i.e. subgroup vocal rates
- `subgr.size`: average number of individuals within 10m during a given time block
- `focal`: two letter code of focal individual
- `focal.grunt` and `focal.other`: whether or not focal produced a vocalization in a given time block
- `asso`: number of associated species
- `otherspec`: vocal rate of non-mangabeys
- `year`, `month`, `day`, `hour`, `minute`, `dt`: time and date of observation
- `sex`: sex of focal individual (male=1, female=0)
- `ac`: auto-correlation term
- `act.fefo`, `act.trvl` and `act.soc`: activity descriptor for each time block (see manuscript for definitions)

```
# packages used to calculate models:
library(lme4); library(effects); library(optimx)
```

```
## Loading required package: Matrix
```

```
packageVersion("lme4")
```

```
## [1] '1.1.11'
```

Model 1 - current speed (dataset1.csv)

```r
xdata <- read.csv("dataset1.csv")
```

```r
full <- lmer(speed ~ subgr.size*(focal.grunt +focal.other +subgr.twit +subgr.grunt +subgr.other)
            +asso*otherspec +sex +ac +(1|focal) +(1|dt), data=xdata, REML = F)
null <- lmer(speed ~ ac +sex +(1|focal) +(1|dt), data=xdata, REML = F)
anova(null, full)
```

```
## Data: xdata
## Models:
## null: speed ~ ac + sex + (1 | focal) + (1 | dt)
## full: speed ~ subgr.size * (focal.grunt + focal.other + subgr.twit +
## full:     subgr.grunt + subgr.other) + asso * otherspec + sex + ac +
## full:     (1 | focal) + (1 | dt)
##      Df    AIC    BIC  logLik deviance  Chisq Chi Df Pr(>Chisq)
## null  6 407.72 426.71 -197.86   395.72
## full 20 399.13 462.42 -179.56   359.13 36.592     14  0.0008501 ***
## ---
## Signif. codes:  0 '***' 0.001 '**' 0.01 '*' 0.05 '.' 0.1 ' ' 1
```

```r
final <- lmer(speed ~ subgr.size*subgr.other +focal.grunt +focal.other +subgr.twit +subgr.grunt
            +asso*otherspec +sex +ac +(1|focal) +(1|dt), data=xdata, REML = F)
coefficients(summary(final))
```

```
##                            Estimate Std. Error      t value
## (Intercept)            -0.312211050 1.27302033  -0.24525221
## subgr.size             -0.047697429 0.04807726  -0.99209961
## subgr.other             0.099798659 0.04615938   2.16204493
## focal.gruntyes         -0.142383359 0.10688397  -1.33213011
## focal.otheryes         -0.049201135 0.13243075  -0.37152350
## subgr.twit             -0.108497350 0.05056477  -2.14571051
## subgr.grunt            -0.067842967 0.04905056  -1.38312319
## asso                   -0.004567669 0.07721705  -0.05915363
## otherspec              -0.028871499 0.05483565  -0.52650965
## sex                     3.990966336 2.27482769   1.75440380
## ac                     -2.610533827 0.14123631 -18.48344719
## subgr.size:subgr.other -0.139770046 0.04099140  -3.40974054
## asso:otherspec          0.100627101 0.04977928   2.02146566
```

Model 2 - future speed (dataset2.csv)

```r
xdata <- read.csv("dataset2.csv")
```

```r
full <- lmer(speed ~ subgr.size*(focal.grunt +focal.other +subgr.twit +subgr.grunt +subgr.other)
            +asso*otherspec +sex +ac +controlspeed +(1|focal) +(1|dt), data=xdata, REML = F)
null <- lmer(speed ~ ac +sex +controlspeed +(1|focal) +(1|dt), data=xdata, REML = F)
anova(null, full)
```

```
## Data: xdata
```

```
## Models:
## null: speed ~ ac + sex + controlspeed + (1 | focal) + (1 | dt)
## full: speed ~ subgr.size * (focal.grunt + focal.other + subgr.twit +
## full:     subgr.grunt + subgr.other) + asso * otherspec + sex + ac +
## full:     controlspeed + (1 | focal) + (1 | dt)
##      Df    AIC    BIC  logLik deviance  Chisq Chi Df Pr(>Chisq)
## null  7 394.32 415.57 -190.16   380.32
## full 21 395.72 459.49 -176.86   353.72 26.599     14     0.0217 *
## ---
## Signif. codes:  0 '***' 0.001 '**' 0.01 '*' 0.05 '.' 0.1 ' ' 1
```

```
final <- lmer(speed ~ subgr.size*subgr.grunt +focal.grunt +focal.other +subgr.twit +subgr.other
            +asso +otherspec +sex +ac +controlspeed +(1|focal) +(1|dt), data=xdata, REML = F)
coefficients(summary(final))
```

```
##                           Estimate Std. Error     t value
## (Intercept)             0.19609079 0.69488197   0.2821929
## subgr.size             -0.03389037 0.06386307  -0.5306725
## subgr.grunt             0.14762172 0.06246476   2.3632801
## focal.gruntyes         -0.10652299 0.14321001  -0.7438236
## focal.otheryes         -0.27995277 0.16943302  -1.6522917
## subgr.twit             -0.02617477 0.06439339  -0.4064822
## subgr.other            -0.15376936 0.06157040  -2.4974558
## asso                   -0.05065415 0.10364668  -0.4887194
## otherspec              -0.01415996 0.06806551  -0.2080343
## sex                     0.52446698 1.24220762   0.4222056
## ac                     -1.60360912 0.12749602 -12.5777192
## controlspeed            0.01846482 0.05676961   0.3252589
## subgr.size:subgr.grunt -0.13027353 0.05545413  -2.3492124
```

Model 3 - current change points (dataset3.csv)

```
xdata <- read.csv("dataset3.csv")
```

```
full <- glmer(cp ~ subgr.size*(focal.grunt +focal.other +subgr.twit +subgr.grunt +subgr.other)
            +asso*otherspec +sex +(1|focal) +(1|dt), data=xdata, family = binomial,
            control=glmerControl(optimizer="optimx", optCtrl=list(method="bobyqa")))
null <- glmer(cp ~ sex +(1|focal) +(1|dt), xdata, family = binomial,
              control=glmerControl(optimizer="optimx", optCtrl=list(method="bobyqa")))
anova(null, full)
```

```
## Data: xdata
## Models:
## null: cp ~ sex + (1 | focal) + (1 | dt)
## full: cp ~ subgr.size * (focal.grunt + focal.other + subgr.twit + subgr.grunt +
## full:     subgr.other) + asso * otherspec + sex + (1 | focal) + (1 |
## full:     dt)
##      Df    AIC    BIC  logLik deviance  Chisq Chi Df Pr(>Chisq)
## null  4 181.36 194.02 -86.681   173.36
## full 18 189.01 245.97 -76.505   153.01 20.353     14     0.1194
```

```r
coefficients(summary(full))
```

```
##                            Estimate Std. Error     z value     Pr(>|z|)
## (Intercept)             -1.67885982  0.3435108 -4.88735737 1.021985e-06
## subgr.size              -0.78173686  0.3076331 -2.54113347 1.104937e-02
## focal.gruntyes          -0.20234145  0.4916924 -0.41152038 6.806910e-01
## focal.otheryes           0.96938133  0.6487447  1.49424169 1.351125e-01
## subgr.twit               0.14046285  0.2748033  0.51113952 6.092534e-01
## subgr.grunt              0.01363847  0.2591253  0.05263270 9.580246e-01
## subgr.other              0.01841090  0.2499868  0.07364749 9.412909e-01
## asso                    -0.14067453  0.3136677 -0.44848262 6.538049e-01
## otherspec                0.31693546  0.2673528  1.18545789 2.358365e-01
## sex                      0.04840826  0.6068623  0.07976811 9.364217e-01
## subgr.size:focal.gruntyes  0.21615621  0.6027842  0.35859631 7.198971e-01
## subgr.size:focal.otheryes  2.33960383  1.0421607  2.24495486 2.477103e-02
## subgr.size:subgr.twit   -0.07968328  0.3038457 -0.26224915 7.931294e-01
## subgr.size:subgr.grunt   0.03559487  0.3018382  0.11792699 9.061255e-01
## subgr.size:subgr.other  -0.56020120  0.2830912 -1.97887205 4.783041e-02
## asso:otherspec           0.36081024  0.2042221  1.76675440 7.726937e-02
```

Model 4 - future change points (dataset4.csv)

```r
xdata <- read.csv("dataset4.csv")
```

```r
full <- glmer(cp ~ subgr.size*(focal.grunt +focal.other +subgr.twit +subgr.grunt +subgr.other)
            +asso*otherspec +sex +controlcp +(1|focal) +(1|dt), data=xdata, family = binomial,
            control=glmerControl(optimizer="optimx", optCtrl=list(method="bobyqa")))
null <- glmer(cp ~ sex +controlcp +(1|focal) +(1|dt), xdata, family = binomial,
              control=glmerControl(optimizer="optimx", optCtrl=list(method="bobyqa")))
anova(null, full)
```

```
## Data: xdata
## Models:
## null: cp ~ sex + controlcp + (1 | focal) + (1 | dt)
## full: cp ~ subgr.size * (focal.grunt + focal.other + subgr.twit + subgr.grunt +
## full:     subgr.other) + asso * otherspec + sex + controlcp + (1 |
## full:     focal) + (1 | dt)
##      Df    AIC    BIC  logLik deviance  Chisq Chi Df Pr(>Chisq)
## null  5 164.01 179.19 -77.004   154.01
## full 19 168.64 226.34 -65.321   130.64 23.365     14    0.05458 .
## ---
## Signif. codes:  0 '***' 0.001 '**' 0.01 '*' 0.05 '.' 0.1 ' ' 1
```

```r
final <- glmer(cp ~ subgr.size*subgr.other +focal.grunt +focal.other +subgr.twit +subgr.grunt
            +asso +otherspec +sex +controlcp +(1|focal) +(1|dt), data=xdata, family = binomial,
            control=glmerControl(optimizer="optimx", optCtrl=list(method="bobyqa")))

coefficients(summary(final))
```

```
##                           Estimate Std. Error    z value     Pr(>|z|)
## (Intercept)            -1.76086336  0.3629391 -4.8516771 1.224218e-06
## subgr.size             -0.42770543  0.2537608 -1.6854670 9.189850e-02
## subgr.other            -0.32255335  0.2485636 -1.2976695 1.944009e-01
## focal.gruntyes          0.45814579  0.4821099  0.9502933 3.419632e-01
## focal.otheryes          0.49891764  0.6349296  0.7857843 4.319939e-01
## subgr.twit              0.49425701  0.2545788  1.9414697 5.220133e-02
## subgr.grunt            -0.09538229  0.2409274 -0.3958964 6.921815e-01
## asso                   -0.30568344  0.2515607 -1.2151480 2.243096e-01
## otherspec              -0.02677187  0.2277026 -0.1175738 9.064053e-01
## sex                     0.71231229  0.6101830  1.1673748 2.430590e-01
## controlcp               1.31457447  0.4759048  2.7622634 5.740215e-03
## subgr.size:subgr.other -0.55925470  0.2876415 -1.9442767 5.186210e-02
```
